# Time-modulated 1-bit amplitude-coded metasurface for space-frequency beam shaping

**DOI:** 10.1038/s41598-025-95415-x

**Published:** 2025-03-31

**Authors:** Seyed Ehsan Hosseininejad, Mohsen Khalily, Rahim Tafazolli

**Affiliations:** Institute for Communication Systems (ICS), Home of the 5G and 6G Innovation Centres, University of Surrey, Guildford, UK

**Keywords:** Adaptive optics, Applied physics

## Abstract

Two-dimensional (2D) space-encoded metamaterials enable the progressive development of a multitude of functionalities for controlling electromagnetic (EM) waves. Recently, time-modulated phase-coded metasurfaces have been introduced into 2D metamaterials, allowing for EM wave manipulation across both spatial and frequency domains. This approach is naturally suited to microwave frequency range where the compact phase controlling elements, such as diodes and varactors are readily available. However, as future generation communication systems target frequencies above 100 GHz with a much higher speed and more densification, enhanced solutions utilizing the novel concepts will be inevitable because the metasurface integration with the common active elements is challenging. Here, we propose the space-time 1-bit amplitude-coded metasurfaces instead of phase-coded one to support the complex functionality for the intelligent control of the environment in wireless communication systems. The solution is implemented using graphene-based meta-atom, where 1-bit amplitude-coding is reconfigurable via bias voltage. To validate the effectiveness of this approach for beam shaping, we demonstrate beam steering with low sidelobe patterns by achieving the desired effective amplitude and phase distributions. This proposed method is anticipated to broaden the applications of digital coding metasurfaces, particularly in THz wireless communication systems.

## Introduction

Frequencies above 100 GHz, have been proposed for 6G and beyond as a primary enabler of revolutionary applications demanding ultra-high data rates exceeding tens of Gigabits per second such as wireless communication, imaging, positioning, wireless cognition, and sensing^1,2^. As next communication systems move toward higher frequencies, novel solutions for programmable control of EM waves are required. Metamaterials, artificial materials whose effective constitutive parameters are modulated in space, have been introduced as a powerful tool to manipulate the EM waves in a myriad of applications. In particular, metasurfaces, that are thin two-dimensional metamaterials, have been demonstrated to produce compelling radiation properties to reliably communicate in the next wireless networks^3–7^.

On a parallel line, time-modulated array antennas have maturely demonstrated simple realization of the desired radiation patterns with considering the time dimension as an additional degree of freedom within the antenna community^8^. Indeed, manipulation of the beam pattern is simply performed by the RF switches in the timed arrays instead of considering amplifiers and phase shifters as applied in phased arrays^9,10^. This concept has recently received considerable attention to antenna problems for the new-generation communications^11^. Recently, the idea behind time-modulated array antennas has been offered to design metasurfaces called ”space-time-coding digital metasurfaces” to manipulate their scattering properties in both the space and frequency domains using time-modulated phase coding in GHz frequencies^12^. Realization of this idea is challenging for frequencies above 100 GHz since the required integrated phase controlling elements, such as positive-intrinsic-negative (PIN) diodes and varactors are easily available in microwave frequency range.

On the other hand, as a kind of emerging two-dimensional material, graphene, a one-atom-thick layer of carbon atoms arranged in a hexagonal lattice, has attracted significant interest due to its superior electronic and optical properties^13^. Such thinnest active material are highly desired to integrate with the passive planar metasurface since the response of graphene can be modulated by DC gate biasing in a wide spectrum from sub-THz to mid-infrared. The conductivity modulation of graphene in various sub-bands is different so that the modulation focused on the real part at sub-THz and low THz band and on the imaginary part in the high THz band^14^. The mentioned characteristics turn graphene into a unique tunable material for the implementation of reconfigurable metasurfaces for next generation of communication, particularly for above 100 GHz where the integration with common active elements, such as diodes and varactors are constrained.

**Fig. 1:**
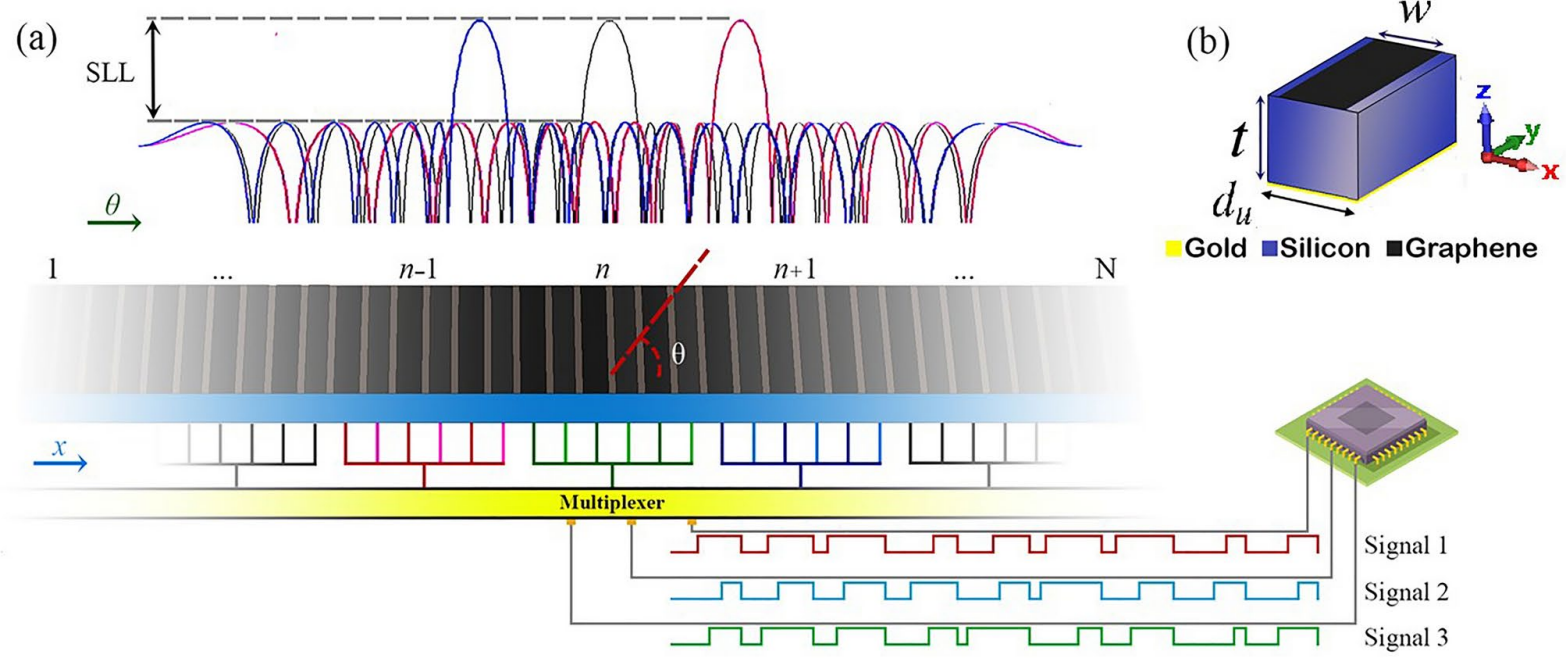
(**a**) Conceptual schematic of the time-modulated amplitude-encoded digital metasurface for beam shaping. (**b**) The graphene-based meta-atom structure with dimensions: $w = 0.13$ mm, $t = 0.185$ mm, and $d_u = 0.17$ mm.

In the theoretical designs and numerical validations, phase modulation in graphene metasurfaces have been applied for high terahertz frequencies (several THz), such as beam focusing^15,16^ and beam steering^17,18^, where surface plasmon (SP) wave supported by graphene. However, since a single graphene layer is not able to provide the required full $360^\circ$ phase range, a stack of graphene layers with dielectric materials as separators was used in the metasurface meta-atoms in these works which is complicated to practically implement the necessary independent biasing structures among graphene layers^19^. On the other hand, graphene is not able to provide the required phase modulation in GHz frequencies, sub-THz frequencies, and low frequencies of THz band^14^. In sub-THz, only graphene-based absorbers have been introduced and fabicated, where graphene shows tunable sheet resistance, resulting in tunable reflection properties^20,21^. Therefore, to achieve a more feasible structure with only one graphene layer, here we propose technique of amplitude modulation in space-time-coding digital metasurface based on graphene to equivalently attain the phase modulation in harmonic frequencies for beam steering.

Furthermore, the metasurface structures have shown unnecessary sidelobes radiation in most cases because providing the required amplitude distribution for good sidelobe behavior can not easily satisfied. This issue can be extremely serious since the high sidelobes produce unwanted beams, which are defective for practical implementation. Here, we propose the time-modulated amplitude-encoded metasurface concept to design metasurfaces with not only beam steering ability but also low sidelobe patterns as conceptually shown in Fig. 1a^22^. It is demonstrated that a time-modulated only 1-bit coding meta-atom is perfectly adequate for our goal, while numerous accurate states are required without considering the time dimension for the meta-atoms which is not practical.

## Theory of time-modulated amplitude-coding metasurfaces

Considering a one-dimensional metasurface shown in Fig. 1 with N super meta-atoms whose dimensions are $d_s$, in which each super meta-atom is occupied by a sub-array of meta-atoms, called cluster, the far-field scattering function can be written as^23^

1 $$\begin{aligned} \begin{array}{l} F(\theta ) = \sum \limits _{n = 1}^N {{A_n}{e^{j{\varphi _n}}}{e^{j{\psi _n}}},\,\,\,\,\,\,} \\ \quad {\psi _n} = {2\pi {f_0}}t + (n - 1)kd_s\sin \theta \end{array} \end{aligned}$$

where $A_n$ and $\phi _n$ describe the excitation amplitudes and phases of the clusters, respectively. Moreover, ${f_0}$ is the operating frequency and $k = 2\pi /\lambda _0$ is the wave number. It should be noted that super meta-atoms consist of *c* number of meta-atoms with the dimension of $d_u$. So, the cluster size ($d_s$) can be written as

2 $$\begin{aligned} {d_s} = c{d_u} \end{aligned}$$

Main beam scanning is performed if a proper linear phase progression is assumed between the elements. Thus, if we desire an scattering pattern maximum in the $\theta _0$ direction, the required element-to-element phase shift ($\phi _n$) is $-kdsin\theta _0$. In our previous work^18^, we presented the theory of beam steering in digital metasurfaces where at least 2-bit phase coding meta-atom is required. For realizing multi-bit phase coding, stacking of graphene layers was proposed since single layer is not able to do that. However, implementing separate gate biasing among elements in the stacked layers is practically complicated. Consequently, here, we survey time-modulated amplitude coding instead of the static phase coding to attain beam scanning in the metasurfaces.

Assuming that the $A_n$’s in Eq. (1) are functions of time as periodic pulse functions. The frequency of the pulses ($f _p$) is much smaller than the central frequency of the metasurface ($f _0$). Consequently, if we apply Fourier series of $A_n(t)$ in the equation, the scattering function is rewritten as

3 $$\begin{aligned} \begin{array}{l} F(\theta ) = \sum \limits _{n = 1}^N {\sum \limits _{m = - \infty }^\infty {{a_{mn}}{e^{jm{2\pi {f_p}}t}}{e^{j{\varphi _n}}}\,} {e^{j\left[ {{2\pi {f_0}}t + (n - 1)k{d_s}\sin \theta } \right] }},\,\,} \\ \quad {a_{mn}} = \frac{1}{{{T_p}}}\int \limits _0^T {{A_n}(t){e^{ - jm{\omega _p}t}}dt} , \end{array} \end{aligned}$$

where, $T_p=1/f_p$ is the period of pulse function. Therefore, the time-modulated metasurface radiation at the harmonics frequencies (${f_0} + m {f_p}$) can be expressed by

4 $$\begin{aligned} {F_m}(\theta ) = \,{e^{j({2\pi {f_0}}t + m{2\pi {f_p}}t)}}\,\sum \limits _{n = 1}^N {{a_{mn}}{e^{j{\varphi _n}}}{e^{j\left[ {(n - 1)k{d_s}\sin \theta } \right] }}\,\,} \end{aligned}$$

We consider only 1-bit amplitude coding meta-atom in the time-varying metasurface structure to achieve both the desired equivalent amplitude distribution for SLL controlling and the desired equivalent phase profile for beam steering in the harmonic frequencies. In fact, for the 1-bit coding case, the reflection amplitude of each super meta-atom is periodically switched between the high-amplitude state “1” and the low-amplitude state “0”. For that reason, a periodic pulse function with modulation period $T_p$ is applied to change between two states for the nth element. Therefore, first period of the $A_n$’s are written as

5 $$\begin{aligned} {A_n}(t) = \left\{ \begin{array}{l} 1\,\,\,\,\,\,\,\,\,\,\,{t_{0n}} \le t \le {t_{0n}} + {\tau _n}\\ 0\,\,\,\,\,\,\,\,\,\,\mathrm{{otherwise}} \end{array} \right. \end{aligned}$$

where $t_{0n}$ is the instant time of the ”1” state and $\tau _n$ is the ”1”-state duration of the meta-atom. Thus, the effective amplitudes and phases of meta-atoms in the harmonic of mth are extracted as

6 $$\begin{aligned} {a_{mn}} = \left| {{a_{mn}}} \right| {e^{j\angle {a_{mn}}}} = \frac{{{\tau _n}}}{{{T_p}}}\frac{{\sin (m\pi \frac{{{\tau _n}}}{{{T_p}}})}}{{m\pi \frac{{{\tau _n}}}{{{T_p}}}}}{e^{ - jm\pi \left( \frac{{2{t_{0n}}}}{{{T_p}}} + \frac{{{\tau _n}}}{{{T_p}}}\right) }}\end{aligned}$$

Consequently, we can design the equivalent amplitude and phase profiles for the metasurface at specified harmonic frequencies. To explain more, consider the scattering functions at the center frequency and the 1th harmonic frequencies which are expressed by

7 $$\begin{aligned} \begin{array}{*{20}{l}} {{F_0}(\theta ) = {e^{j2\pi {f_0}t}}\sum \limits _{n = 1}^N {\frac{{{\tau _n}}}{{{T_p}}}{e^{j\left[ {{\varphi _n} + (n - 1)k{d_s}\sin \theta } \right] }}{\hspace{0.55542pt}} {\hspace{0.55542pt}} } ,}\\ \begin{array}{l} {F_1}(\theta ) = {e^{j(2\pi {f_0} + 2\pi {f_p})t}}\sum \limits _{n = 1}^N \begin{array}{l} \frac{{\sin \left( \pi \frac{{{\tau _n}}}{{{T_p}}}\right) }}{\pi }{e^{ - j\pi \left( \frac{{2{t_{0n}}}}{{{T_p}}} + \frac{{{\tau _n}}}{{{T_p}}}\right) }}\\ {e^{j\left[ {{\varphi _n} + (n - 1)k{d_s}\sin \theta } \right] }}{\hspace{0.55542pt}} {\hspace{0.55542pt}} \end{array} \\ {F_{ - 1}}(\theta ) = {e^{j(2\pi {f_0} - 2\pi {f_p})t}}\sum \limits _{n = 1}^N \begin{array}{l} \frac{{\sin \left( \pi \frac{{{\tau _n}}}{{{T_p}}}\right) }}{\pi }{e^{ + j\pi \left( \frac{{2{t_{0n}}}}{{{T_p}}} + \frac{{{\tau _n}}}{{{T_p}}}\right) }}\\ {e^{j\left[ {{\varphi _n} + (n - 1)k{d_s}\sin \theta } \right] }}{\hspace{0.55542pt}} {\hspace{0.55542pt}} \end{array} \\ \end{array} \end{array} \end{aligned}$$

${F_0}$ shows that $\tau _n$ can manipulate the effective amplitude of meta-atoms at the center frequency ($\left| {{a_{0n}}} \right|$)) to realize the required amplitude distribution. For instance, Dolph-Chebyshev pattern can be considered to provide highest directivity and lowest beamwidth for a given sidelobe level. However, we can not perform beam steering at the center frequency using only time-modulated amplitude coding since this function requires phase manipulation of meta-atoms. While ${F_1}$ and ${F_{-1}}$ demonstrate that it is possible to change the main beam direction $\theta _0$ at the first positive sideband via a suitable equivalent phase progression ($\angle {a_{1n}}$) which is achieved by setting $\frac{{{t_{0n}}}}{{{T_p}}}$ as

8 $$\begin{aligned} \frac{{{t_{0n}}}}{{{T_p}}} = \frac{1}{{2\pi }}\left( {{\varphi _n} + j\left[ {(n - 1)kd\cos {\theta _0}} \right] } \right) - \frac{1}{2}\frac{{{\tau _n}}}{{{T_p}}}\ \end{aligned}$$

where $\phi _n$ is constant for all meta-atoms. On the other hand, $\tau _n$ can control the effective amplitude of meta-atoms ($\left| {{a_{1n}}} \right|$) so that according to the desired amplitude tapering, $\frac{{{\tau _n}}}{{{T_p}}}$ is extracted as

9 $$\begin{aligned} \frac{{{\tau _n}}}{{{T_p}}} = \frac{1}{\pi }{\sin ^{ - 1}}(\pi \left| {{a_{1n}}} \right| )\ \end{aligned}$$

Consequently, a reconfigurable meta-atom with 1-bit amplitude quantization (0/1) is perfectly adequate for our needs i.e. control of effective amplitude and phase of meta-atoms at harmonic frequencies although the required number of bits for the digital amplitude-phase-modulated metasurface without time modulation is much higher than one.

## 1-Bit amplitude-coding meta-atom structure

In the following, the desired meta-atom i.e. 1-bit amplitude-modulated coding meta-atom based on graphene for the frequencies above 100 GHz will be presented. According to the Kubo formula, the complex surface conductivity of graphene in the low THz frequency regime can be expressed as^24^

10 $$\begin{aligned} \begin{aligned}&\sigma _{\text {graphene}}\left( \omega , \mu _{c}, \Gamma , T\right) \\&\quad =-j \frac{e^{2} k_{B} T}{\pi \hbar ^{2}(\omega -j 2 \Gamma )}\left( \frac{\mu _{c}}{k_{B} T}+2 \ln \left( e^{-\mu _{c} / k_{B} T}+1\right) \right) . \end{aligned} \end{aligned}$$

where, *e* is the electron charge, $\hbar =h/(2\pi )$ is the reduced Planck’s constant, $k_b$ is the Boltzmann’s constant, $\Gamma =1/(2\tau )$ is the electron scattering rate, and $\tau$ is the transport relaxation time. The chemical potential of graphene monolayer is a function of external bias, which can be adjusted dynamically via applying a proper DC voltage source. This paper considers the temperature and relaxation time as $T=300$ K and $\tau =0.04$ ps. The selected value for the relaxation time is practical, which is the most important advantage of this design.

In order to design the reconfigurable device, a graphene monolayer is embedded in the meta-atom as depicted in Fig. 1b. The meta-atom consists of sub-wavelength patterned graphene ribbons with widths of *w* on a spacing silicon layer with a thickness of $t=$ terminated by a gold layer. In addition, the period of the meta-atom is $d_u$.

According to the theoretical framework described in the section “Introduction”, our design goal include achieving two distinct reflection amplitude states-high (state “1”) and low (state “0”)-at frequencies above 100 GHz, specifically centered at 118 GHz. By carefully engineering the ribbon width and other meta-atom dimensions, we optimize the meta-atom to exhibit tailored reflection amplitudes. This design choice enables dynamic modulation of reflection properties, allowing the meta-atom to switch efficiently between these two states. Therefore, the dimensions of meta-atom are meticulously selected as follows: $w=0.13$ mm, $t=0.185$ mm, and $d_u = 0.17$ mm.

**Fig. 2:**
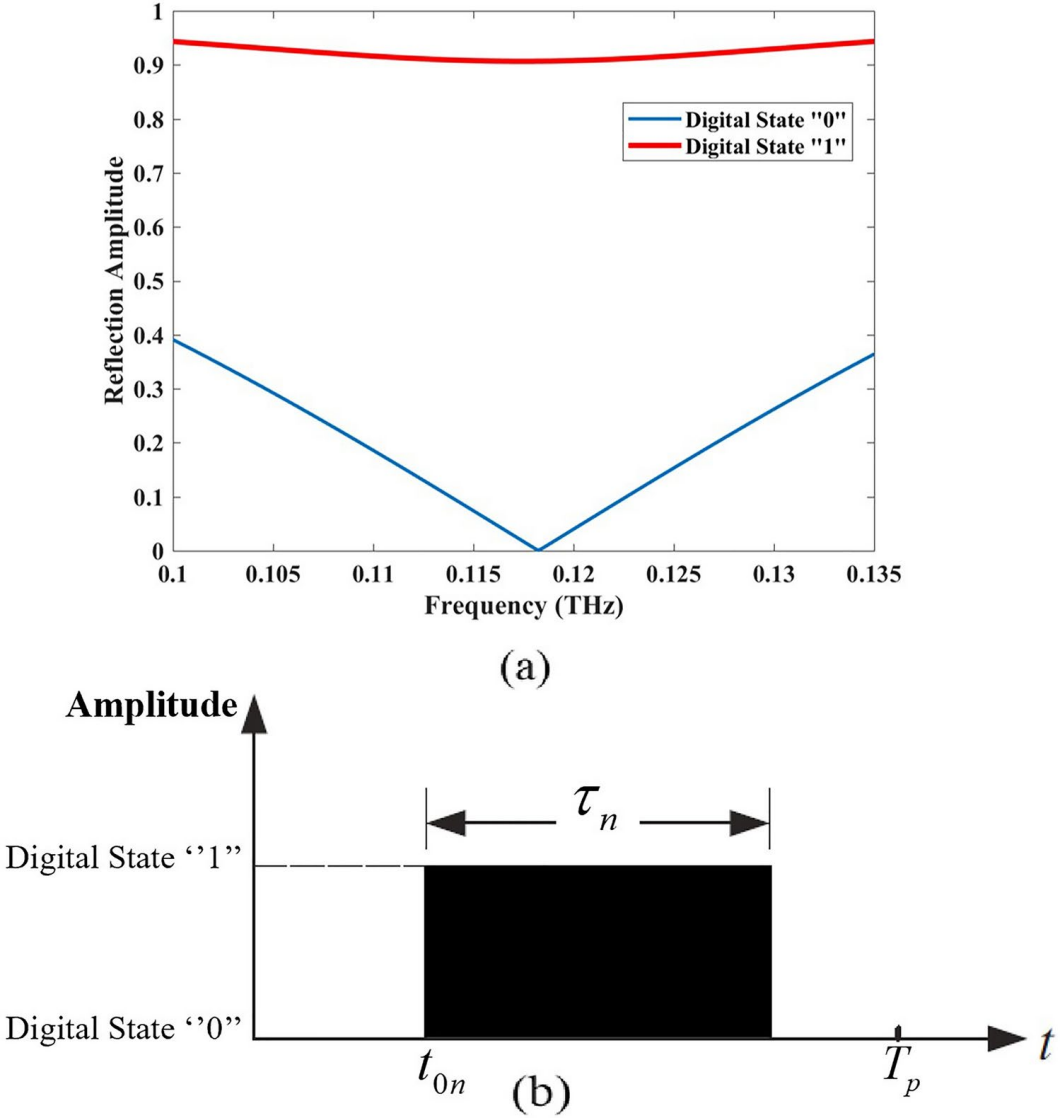
Reflection amplitude of the 1-bit meta-atom which show 2 states at 118 GHz with changing chemical potentials of graphene layers, (**b**) time function of the nth element.

The meta-atom structure is simulated in CST Studio Suite to obtain the responses of reflection for y-polarized incident waves. The coordinate system is shown in Fig. 1b. Figure 2a shows the simulated reflection amplitudes for various chemical potentials of the graphene ribbon. The reflection states are chosen with two different states of “0” and “1”. The value of chemical potential is selected as 0.74 eV and 0 eV for “0” and “1” states as they switch to each other according to the time function shown in Fig. 2b.

To assess the operational bandwidth, we define the effective bandwidth as the frequency range over which a clear distinction between the two reflection amplitude states is maintained. As depicted in Fig. 1a, the reflection amplitude for State “0” remains below 0.316 (− 10 dB) within the frequency range of 0.104 THz to 0.132 THz, indicating strong attenuation in this state. On the other hand, state “1” maintains a relatively stable reflection amplitude above 0.9 across the analyzed frequency band, ensuring high reflection and contrast between the two states. This distinct amplitude difference over a   28 GHz bandwidth highlights the suitability of this approach for broadband applications.

The graphene ribbons are biased easily from sides with the predetermined voltage layout. In the proposed structure, an Al2O3 layer is sandwiched between the graphene monolayer and a thin doped silicon substrate to implement the meta-atom. A doped silicon thin layer and graphene ribbon are enough to bias meta-atom from one side. The required DC voltage can be applied to each ribbon via metallic contact. In the proposed scheme, gold contact is connected to the thin silicon layer, and chromium contacts are connected to the graphene ribbons. An external DC voltage can be used to control graphene ribbons’ chemical potential dynamically. In the proposed biasing scheme, a biasing voltage can be obtained by using a parallel plate capacitor as^25^

11 $$\begin{aligned} V_{DC}=\frac{\mu _c^2h_{Al2O3}e}{\hbar ^2v_F^2\pi \varepsilon _0\varepsilon _{Al2O3}}. \end{aligned}$$

In the above equation, $v_F = 10^6$ m/s is the Fermi velocity, $\varepsilon _0$ is vacuum permittivity, $\varepsilon _{Al2O3}$ is the relative permittivity, and $h_{Al2O3}$ is the thickness of the Al2O3 thin layer. By choosing a proper biasing voltage and applying this value via an external processor, we can generate the required chemical potential for each graphene ribbon.

## Results and discussion

In this section, we exemplify the design process of the proposed metasurfaces. The design approach focuses on achieving complex beam shaping, here controlling beam direction with low sidelobe levels by combining both spatial and temporal amplitude-coding strategies. The designed metasurface consists of an array of 200 meta-atoms, which are divided into 40 clusters, with each cluster containing 5 meta-atoms.

### Low sidelobe radiation using time-modulated 1-bit amplitude coding metasurface at central frequency

Considering the amplitude coefficients derived using the Dolph-Chebyshev technique for a sidelobe level (SLL) of -30 dB, the amplitude profile is discretized into 1-bit (2 states) and 3-bit (8 states) quantization levels to implement a metasurface that achieves a low sidelobe radiation pattern at the central frequency. This discretization allows for practical implementation in digital coding metasurfaces, where the number of available states directly influences the ability to replicate the ideal amplitude distribution.

**Fig. 3:**
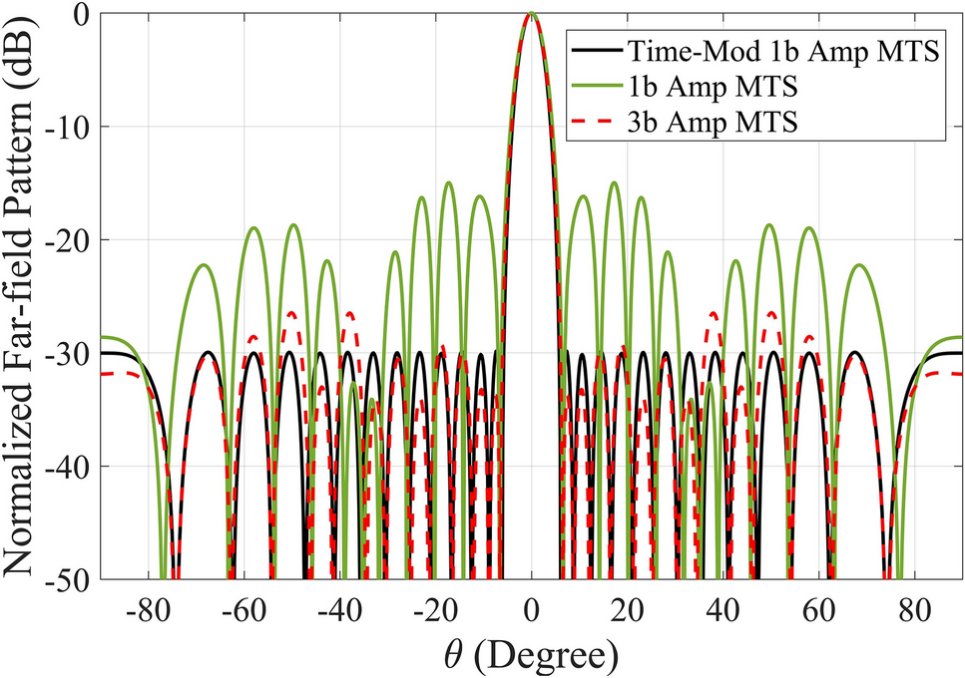
The normalized far-field patterns of the metasurface to achieve SLL = − 30 dB, 1-bit amplitude metasurface, 3-bit amplitude metasurface, and time-modulated 1-bit amplitude metasurface.

The calculated far-field radiation patterns (Fig. 3) indicate that increasing the number of digital states per meta-atom results in a closer approximation to the ideal Dolph-Chebyshev radiation pattern. Specifically, metasurfaces with a higher number of quantization states (such as the 3-bit case) offer improved accuracy in terms of achieving the desired low sidelobe levels. However, the trade-off is that higher quantization levels require more complex meta-atom structures, which can introduce design challenges and increased fabrication costs.

**Fig. 4:**
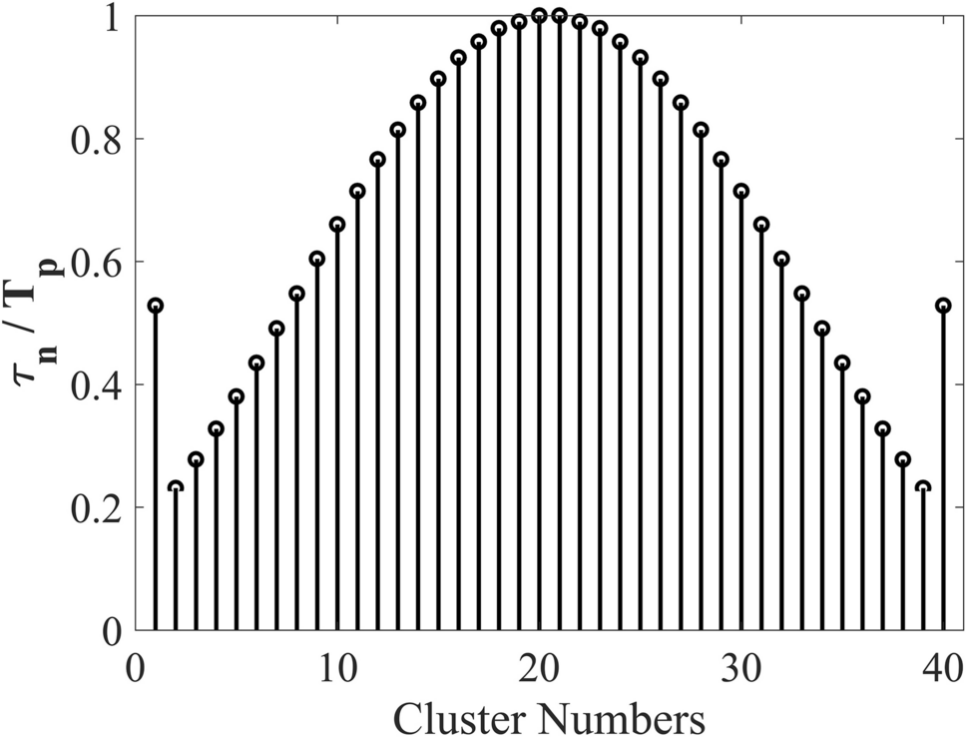
Time sequences of cluster states of meta-atoms ($\frac{{{\tau _n}}}{{{T_p}}}$) for the time-modulated 1-bit metasurface to achieve SLL = − 30 dB.

To address this issue, we propose the use of a time-modulated amplitude-coded metasurface that requires only two digital states (1-bit) per meta-atom. By carefully applying time modulation, we can effectively synthesize the desired amplitude distribution without the need for increased structural complexity. The time sequences for clusters of meta-atoms in the time-modulated 1-bit metasurface are optimized and extracted, as illustrated in Fig. 4.

The results, shown in Fig. 3, demonstrate that the desired Dolph-Chebyshev pattern is achieved with remarkable accuracy, even with just two digital states. This confirms that time modulation can successfully compensate for the limited number of amplitude states, allowing the metasurface to maintain low sidelobe levels without adding complexity to the meta-atom design.

### Beam direction and sidelobe control using time-modulated 1-bit amplitude coding metasurface at harmonic frequencies

**Fig. 5:**
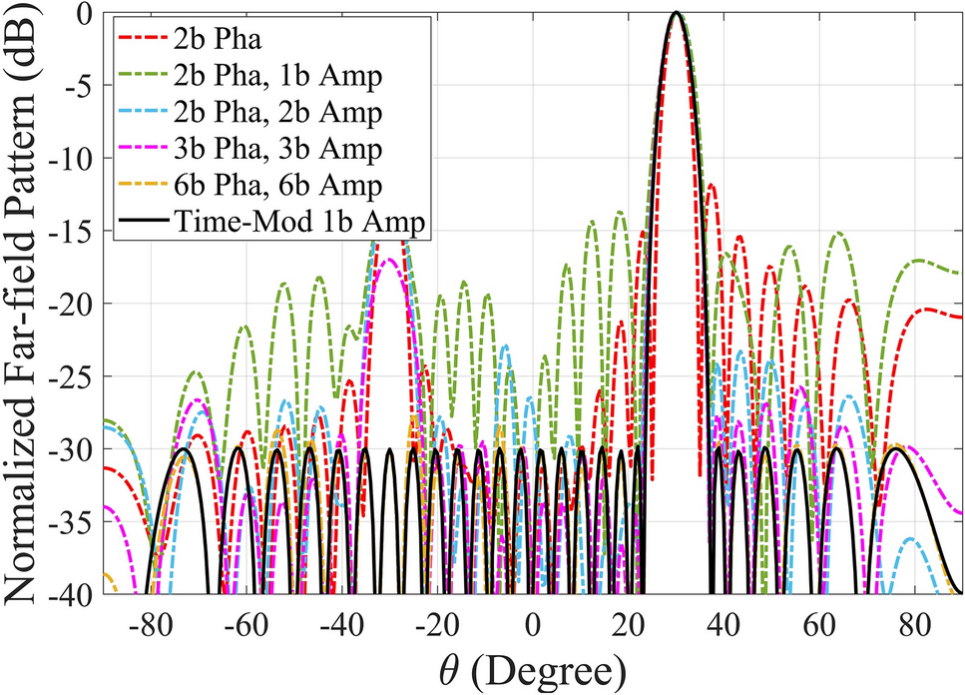
The normalized far-field patterns of the metasurface to achieve $\theta _0=30^{\circ }$ and SLL = − 30 dB, comparing our approach (time-modulated 1-bit amplitude coding) with other approaches (2-bit phase coding, 2-bit phase and 1-bit amplitude coding, 2-bit phase and 2-bit amplitude coding, 3-bit phase and 3-bit amplitude coding, 6-bit phase and 6-bit amplitude coding).

**Fig. 6:**
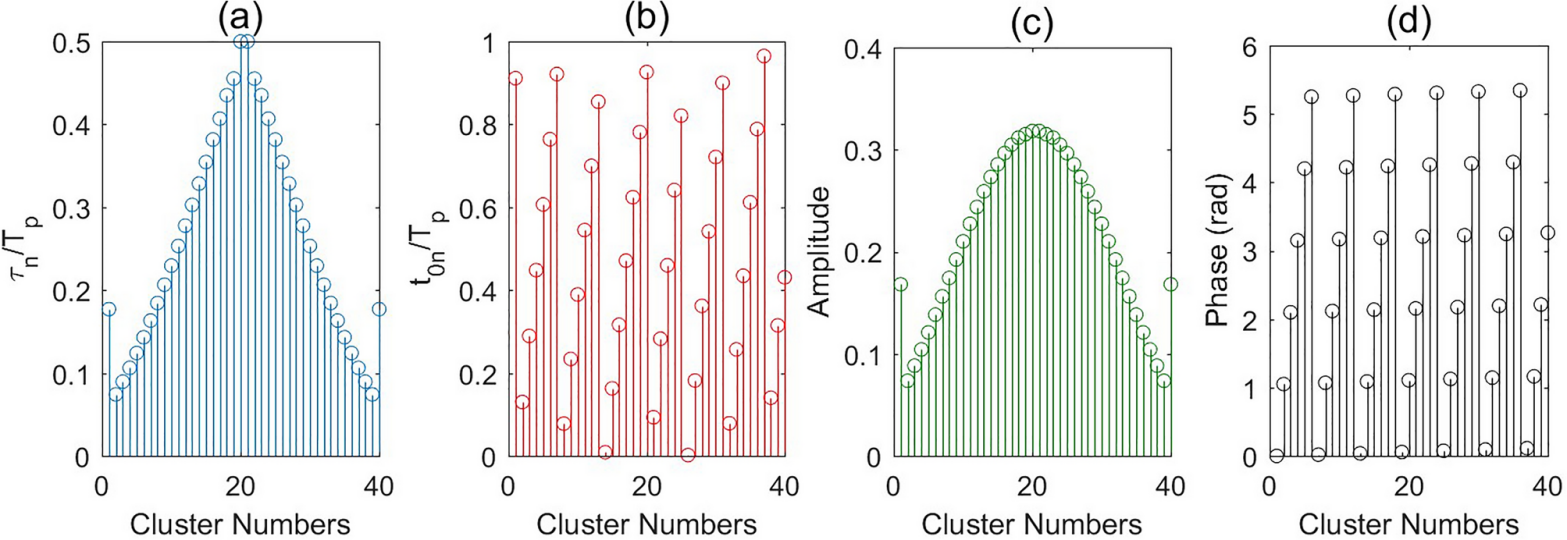
(**a**, **b**) Time sequences of cluster states of meta-atoms ($\frac{{{\tau _n}}}{{{T_p}}}$ and $\frac{{{t_{0n}}}}{{{T_p}}}$), (**c**, **d**) Equivalent amplitude and phase for the time-modulated 1-bit metasurface to achieve $\theta _0=30^{\circ }$ and SLL = − 30 dB at first harmonic frequency.

This section will utilize the time-modulation to manipulate the reflected beam in the desired direction. Moreover, we can control the SLL dynamically by using the proposed method. As an example, the reflected beam is redirected to $\theta _0=30^{\circ }$ whereas SLL $=-30$ dB. In order to demonstrate the advantage of the time-modulated metasurface, we compare its far-field result with the static ones without time dimension. First, let us consider the static metasurface with the 2-bit encoded phase. The calculated far-field pattern for this case is illustrated in Fig. 5. As we can observe in this plot, the main beam is redirected to the desired direction, but the side lobes behavior is not acceptable. So far, the uniform amplitude is considered for all the elements. Next, an amplitude control is added to the metasurface to modify the side lobes. In the next cases, 2-bit phase and 1-bit amplitude, 2-bit phase and 2-bit amplitude, 3-bit phase and 3-bit amplitude, and 6-bit phase and 6-bit amplitude are used to manipulate the wave. The calculated results for the mentioned cases are shown in Fig. 5. As we can observe in this plot, a precise redirected angle is obtained with 2-bit phase coding; however, increasing the states of amplitude profile to elements decreases the SLL value. In other words, according to the obtained result, 2-bit phase control is enough to manipulate the wave into the desired direction while more amplitude states are necessary to get the desired SLL value, here 6-bit amplitude control, which requires a complex structure. Finally, we use the time-modulation 1-bit amplitude coding approach to perform beam shaping. The pulse duration and initial time are calculated and illustrated in Fig. 6a and b, respectively. The equivalent amplitude and phase at first harmonic frequency for this example are shown in Fig. 6c and d.The calculated far-field pattern shows excellent agreement with our desired reflection angle and SLL value. To elaborate further, 6-bit phase and 6-bit amplitude coding amplitude achieves the desired beam shaping ($\theta _0=30^{\circ }$ and SLL = − 30 dB), while the time-modulated 1-bit approach also reaches this target. This highlights that time modulation can compensate for the limitations of 1-bit coding, achieving comparable performance to multi-bit coding with significantly reduced implementation complexity. Therefore, the results emphasize that the time-modulated 1-bit approach provides an optimal trade-off between performance and implementation feasibility, making it a practical choice for beam forming applications.

The presented example demonstrates that we can manipulate the wave in space with the desired parameters by periodically switching the meta-atoms between only two amplitude states, which is not easily achievable with meta-atoms without time dimension, even those with multiple states. Furthermore, the periodicity of the time modulation determines the frequency spectrum of the scattered wave. As a result, the time-modulated amplitude-coded metasurfaces enables dynamic control over both the spatial and spectral characteristics of the reflected beams, achieving flexible space-frequency beam shaping.

The feasibility of realizing the proposed metasurface follows a well-established fabrication protocol, as outlined in^26,27^. Initially, a monolayer of graphene is synthesized via chemical vapor deposition (CVD) on a copper foil. This graphene layer is subsequently transferred onto a dielectric spacer consisting of a 65 nm-thick alumina (Al$_2$O$_3$) layer, uniformly deposited onto an ultrahigh-resistivity p-type polycrystalline wafer via atomic layer deposition. Following the transfer onto a silicon wafer, precise patterning is achieved using 100 keV electron beam lithography with Polymethyl methacrylate (PMMA) as the resist, followed by oxygen plasma etching to remove excess graphene, ensuring the formation of ribbon regions of predetermined dimensions. Given that the auxiliary layers are significantly thinner than the operational wavelength, their impact on reflection remains negligible. Finally, the bottom wafer can be detached, and a metallic ground plane can be deposited onto the substrate layer. Moreover, it is noted that the selected relaxation time for graphene is chosen to be practical, ensuring feasibility in real-world applications.

Consequently, the proposed approach, compared to conventional phase-modulated metasurfaces, demonstrates efficient technique across key metrics, including beamforming efficiency, spectral bandwidth, and reconfigurability. It enables advanced and efficient beamforming, overcoming the limitations of traditional phase-coded techniques in the THz frequencies. Additionally, conventional designs struggle to achieve a broad spectral bandwidth, whereas time-modulated 1-bit amplitude coding significantly enhances adaptability across a wide THz range. Furthermore, graphene offers one of the fastest response times among reconfigurable materials, making it highly suitable for real-time tunable THz metasurfaces.

## Conclusion

In conclusion, a time-modulated amplitude-coding approach is proposed for designing metasurfaces that offer advanced performance. This approach achieves the desired beam shaping by redistributing the two amplitude states across both the spatial and temporal domains. The concept is validated using graphene-based meta-atoms, demonstrating beam steering with low sidelobe radiation. These features highlight the breakthrough potential of time-modulated digital metasurface technology, which is expected to play a fundamental role in future-generation communication systems.

## Data Availability

The datasets generated during and/or analyzed during the current study are available from the corresponding author on reasonable request.
